# Magnitude of screening for gestational diabetes mellitus in an urban setting in Tanzania; a cross-sectional analytic study

**DOI:** 10.1186/s12884-020-03115-3

**Published:** 2020-07-23

**Authors:** Akampa Mukuve, Mariam Noorani, Ibrahim Sendagire, Miriam Mgonja

**Affiliations:** 1 Department of Family Medicine, Post Graduate Medical Education, Aga Khan University, Dar es Salaam, Tanzania; 2 Department of Obstetrics and Gynecology, Aga Khan University, P.O BOX 38129, Plot 42, Ufukoni Road, Dar es Salaam, Tanzania; 3 MSF/Epicentre Mbarara Research Centre, Mbarara, Uganda; 4 Department of Pediatrics and Child Health, Aga Khan University, Dar es Salaam, Tanzania

**Keywords:** Tanzania, Hyperglycaemia in pregnancy, Screening

## Abstract

**Background:**

Medical screening detects risk factors for disease or presence of disease in otherwise well persons in order to intervene early and reduce morbidity and mortality. During antenatal care (ANC) it is important to detect conditions that complicate pregnancy, like gestational diabetes mellitus (GDM). Despite international and local guidelines recommending screening for GDM during ANC, there is evidence to suggest that the practice was not being carried out adequately. A major challenge may be lack of consensus on uniform GDM screening and diagnostic guidelines internationally and locally.

The primary objective was to determine the magnitude of screening for GDM among women receiving ANC at the Aga Khan Hospital, Dar es Salaam and Muhimbili National Hospital, Dar es Salaam. Secondary objectives were: to determine the methods used by health practitioners to screen for GDM, to determine the magnitude of undiagnosed gestational diabetes mellitus among women attending ANC and factors associated with screening for GDM among these women.

**Methods:**

A cross-sectional analytical study was done. Data collection was done using pre-tested questionnaires and reviewing antenatal care records. The proportion of women attending ANC who were screened for GDM was determined. The 75 g Oral Glucose Tolerance Test (OGTT) was offered to women who had not been screened after education and consent.

**Results:**

Only 107 out of 358 (29.9%) had been offered some form of GDM screening. Tests used for GDM screening were random blood sugar (56.8%), fasting blood sugar (32.8%), HbA1C (6%) and 75 g OGTT (3.4%). The uptake of the OGTT was 27%. Of these women the prevalence of GDM was 27.9%. Factors associated with screening for GDM were history of big baby, history of pregnancy induced hypertension and participant awareness of GDM (all *p*: < 0.05).

**Conclusions:**

Screening for GDM among women attending ANC was lower than the World Health Organization target. Efforts should be directed towards promoting GDM screening, increasing awareness about GDM and developing more effective screening methods.

## Background

Medical screening identifies apparently healthy people who have a disease or increased risk of disease [1]. The strategy helps clinicians to detect unrecognized symptomatic and pre-symptomatic disease. Screening for conditions therefore enables early diagnosis and intervention which improves clinical outcomes and reduces costs of health care.

Gestational diabetes mellitus (GDM) is diabetes that is first diagnosed in the second or third trimester of pregnancy that is not clearly either preexisting type 1 or type 2 diabetes [2]. GDM is one of the conditions which should be screened for during antenatal care (ANC). This is because GDM increases risk of maternal and fetal complications during pregnancy [3] and adverse health outcomes in the future [4].

The prevalence of GDM varies across populations, ranging from 10.4 to 25% across the world [5]. Over 90% of cases occur in low and middle income countries [6]. Although there is a lot of literature on the prevalence, management and complications of GDM, little focus has been put on how much GDM screening i.e. the magnitude of GDM screening is actually done. A study done in Uganda, Tanzania and Burkina Faso showed that health care workers did not provide all required services during ANC despite good attendance [7]. It is worth also noting that guidelines for screening for GDM vary across the globe and within countries [8]. In Tanzania the national guidelines [9] vary from the Tanzania diabetic association [10] guidelines and both vary from the World Health Organization (WHO) guidelines [3]. Tests used to screen for GDM in these national guidelines include the oral glucose tolerance test (OGTT), random blood sugar (RBS), fasting blood sugar (FBS) and glycated haemoglobin (HbA1C).

Lack of vigilance to screen for GDM and varying guidelines may be responsible for poor GDM screening practices in low resource settings as shown in Cameroon [11]. From other studies, some factors associated with screening for GDM include: education status/ literacy, husband support, family support, awareness of GDM or its risks, health care providers informing the women about the test and help with household work [12], marital status, women’s autonomy [13], employment status [14], private health insurance status, distance to hospital, journey time to health facility, mode of transport and ability to pay for the service/ financial status [15].

Given the significance of the unfavourable pregnancy outcomes of GDM but paucity of data on how much screening is done for the condition, this study sought to determine the magnitude of screening for GDM among women attending ANC at two tertiary hospitals. The study also determined the tests used for GDM screening during antenatal care, prevalence of undiagnosed GDM and factors associated with screening for GDM.

## Methods

### Design and study site

A cross-sectional analytic study was done at the Aga Khan Hospital Dar es Salaam and Muhimbili National Hospital, Dar es Salaam in Tanzania. The Aga Khan Hospital is a not for profit, tertiary care facility and the biggest private hospital in Tanzania. Its antenatal care clinic attends to averagely 300 women a week. Muhimbili hospital is the national referral hospital providing tertiary care services to referral cases and the population surrounding it. Antenatal care attendance in this hospital averages 1000 women per week.

### Participants

Total sample size was 358. Sample size was determined using a standard formula on the basis of 22% magnitude of screening for GDM from a study in a similar setting [11], 20% precision, 80% power, 5% non-response rate and type I error of 5%. Each of the 2 study sites provided half of the total sample. Convenient, consecutive sampling was used to achieve the required sample.

The study population comprised of pregnant women above 18 years of age, who had already received some antenatal care and who were above the gestational age at which screening for GDM is routinely performed. Included were pregnant women attending the antenatal care specialist clinics, aged 18 years and above, possession of antenatal card, gestational age of 30 or more weeks calculated using menstrual dates or earliest ultra sound scan, and who attended a 1st or 2nd trimester antenatal care visit at the study site. Women were excluded if they had pre-existing self-reported diagnosis of diabetes mellitus, severe illness, mental illness, participated in another study or were taking corticosteroids, antidepressants, diuretics or beta blockers.

### Data collection

During the routine health talks all women were educated about GDM and given details about the study. Informed, written and signed consent was obtained from willing participants. A mini check list confirmed eligibility of participants.

Data collection tools were an interviewer-administered questionnaire and data extraction sheet. Participants were interviewed using a pre-tested, structured questionnaire in the language of choice between Kiswahili and English. The questionnaire identified patient demographic characteristics, risk factors for GDM, socio-economic factors associated with screening for GDM, past obstetric and medical history and tests for glycemic status and related information regarding screening for GDM. Additional information including weight, height, body mass index, blood pressure was obtained and verified from the antenatal cards, patient files and laboratory reports. This information was recorded on the data collection sheet. Where information from the interview and patient records differed, the latter were used.

Women were considered screened for GDM if WHO 2013 guidelines [3], Tanzania Diabetic association guidelines [10] or Tanzania ministry of health guidelines 2017 [9] had been followed. All participants found not to have been screened for GDM were offered the 75 g OGTT. The glucometer SD GlucoNavii® GDH blood glucose monitoring system and SD BIOSENSOR INC lancets were used. This apparatus had a coefficient of variation 5% above 5.55 mmol/l and within standard deviation 4 mg/dl for readings below 5.55 mmol/l. For those with medical insurance, the cost of the test was covered by the insurer. Cash paying clients were offered a discounted price at the Aga Khan hospital and it was offered free of charge for those who could not afford. Interpretation of results was according to WHO 2013 guidelines [3]. Women diagnosed with GDM or pre-diabetic states were informed and referred for appropriate care.

Data were collected on possible determinants of screening for GDM which were either socio-economic factors or risk factors of GDM. The socio-economic factors included: education status/ literacy, marital status, employment status, women’s autonomy, private health insurance status, distance to hospital, journey time to health facility, mode of transport, ability to pay for the service/ financial status, husband support, family support, obstetric history, parity, awareness of GDM or its risks, health care providers informing the women about the test and help with household work.

The risk factors for GDM included: glycosuria, Body Mass Index (BMI) > 25 at time of interview, hypertension in current pregnancy, history of chronic hypertension, history of pregnancy induced hypertension (PIH), history of GDM, history of pre-diabetic states, family history of hypertension, family history of diabetes mellitus, history of multiple pregnancy, history of delivering a macrosomic (**≥**4 kg of weight) baby, clinical or ultrasound diagnosis of large for gestational age in the ongoing pregnancy, history of excessive weight gain (**≥**5 kg) since 18 years of age, race, history of pregnancy loss and grand-multiparity (**≥** 5 pregnancies).

### Data analysis

Data was entered into Microsoft excel 2007 and cleaned. The data was then transferred to SPSS 20 for analysis. We defined “magnitude” of screening for GDM as the proportion of participants attending ANC who had received any test aimed at screening for GDM. Various tests done during screening were categorized and frequencies and percentages for each calculated and recorded. The percentage of participants who had not initially screened but were diagnosed with GDM by OGTT in the study was the magnitude of undiagnosed GDM. Socio-economic determinants of screening for GDM and risk factors for GDM were evaluated for association with screening for GDM using the chi-square test and associated *p*-values. Factors found to be statistically significant by chi square test were further analyzed using logistic regression for possible association with screening for GDM.

## Results

**Fig. 1:**
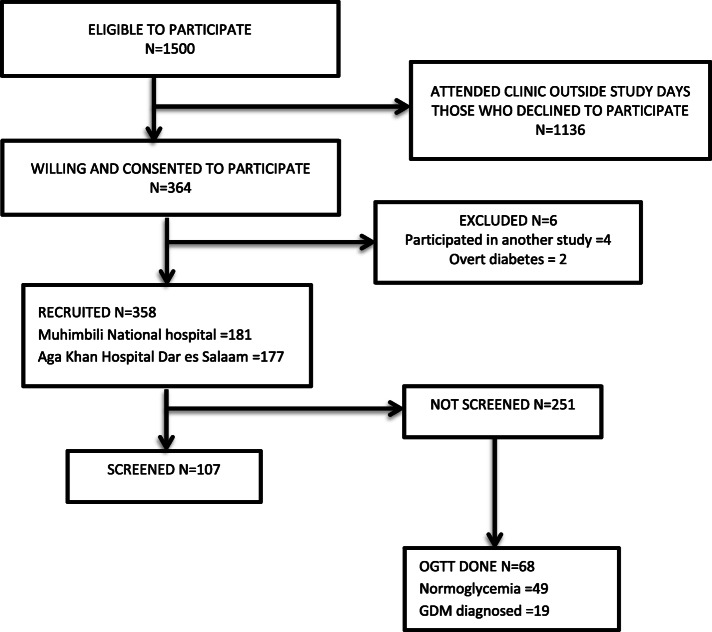
Study profile of participants attending Antenatal Care at Muhimbili National Hospital and Aga Khan Hospital Dar es Salaam in 2018

The study period was July to September 2018. During the study period July to September 2018, there were 2250 women who attended specialist ANC at the study sites. As shown in Fig. 1, approximately 1500 were eligible to participate. Women who consented were interviewed. Six women were excluded. Two had overt diabetes while four had participated in other studies.

Demographic and obstetric characteristics are summarized in Table 1. The mean age of the participants was 30.2 years (SD: 4.8; range 20–44). Most of the participants were between 20 and 30 years, accounting for 57.6% of the study population. The mean gestational age was 34.9 weeks of gestation (SD = 3.1; range 30–42). Of all the participants, 97.2% were black African, while 2.8% were of Asian or of other origins.

**Table 1:** Baseline demographic and clinical characteristics of participants attending Antenatal Care at Muhimbili National Hospital and Aga Khan Hospital Dar es Salaam in 2018

	Frequency (Total 358)	Percentage (Total 100%)
**Age**^a^		
18–25	61	17.1
26–30	135	37.9
31–35	113	31.7
36–40	37	10.4
> 40	10	2.8
**Residence**		
Ilala	95	26.5
Kinondoni	113	31.6
Ubungo	68	19.0
Temeke	42	11.7
Kigamboni	28	7.8
Outside Dar es salaam	12	3.4
**Gravidity**		
Prime Gravida	94	26.3
G2	112	31.3
G3	82	22.9
G4	45	12.6
≥ G5	25	6.9
**Number of GDM risk factors**		
0	2	0.6
1	20	5.6
2	66	18.4
3	114	31.8
4	79	22.1
≥ 5	77	21.5
**Number of previous ANC visits**		
1	10	2.8
2	21	5.9
3	46	12.8
4	78	21.8
5	75	20.9
≥ 6	128	35.8
**Gestational age**		
30–31.9	66	18.4
32–34.9	103	28.8
35–37.9	114	31.9
38–40.9	70	19.5
≥ 41	5	1.39

Key: *ANC* Antenatal Care, ^a^Data missing for 2 participants

### Magnitude of screening for GDM

The overall magnitude of screening for GDM at both hospitals was 29.9% (107 of 358). The magnitude was 21.5% (38 of 177) at Aga Khan Hospital and 38.1% at Muhimbili National Hospital (69 of 181). Of the 251 of women found not to have been screened for GDM only 27% (68) accepted the offered OGTT.

### Tests used to screen for GDM

Of the 107 participants screened for GDM, 56.8% (66) had done random blood sugar (RBS), 32.8% (38) fasting blood sugar (FBS), 6% [7] HbA1C and 3.4% [3] the 75 g Oral glucose tolerance test (OGTT).

### Prevalence of undiagnosed GDM

The prevalence of undiagnosed GDM was 27.9% (19 of 68).

### Factors associated with screening for GDM

As shown in Tables 2 and 3, chi square analysis revealed factors with statistically significant association with screening for GDM to be: awareness about GDM (*p* <  0.001), health worker communication about GDM screening (*p* <  0.001), partner support during ANC (*p* = 0.045), family support during ANC (p 0.049), history of pregnancy induced hypertension (PIH) (*p* = 0.022), history of delivering macrosomic baby (*p* = 0.023) and history of glycosuria (*p* = 0.002). Logistic regression analysis (Table 4) revealed that history of delivering a macrosomic baby (*p* = < 0.001 OR 2.15 95% CI: 1.12–4.10), history of PIH (*p* = < 0.021 OR 2.34 95% CI: 1.13–4.82) and awareness of GDM (*p* = < 0.001 OR 2.79 95% CI: 1.74–4.45) were the only 3 factors associated with screening for GDM.

**Table 2:** Univariable analysis of socio-economic determinants of screening for Gestational Diabetes Mellitus among women attending antenatal clinic at Muhimbili National Hospital and Aga Khan Hospital Dar es Salaam in 2018

Factor	Prevalence (%)	*p*-value
Educated > tertiary level	71.8	0.926
Married	82.4	0.880
Independent source of income	79.6	0.270
Autonomy	76.0	0.331
Medical insurance	71.2	0.412
Personal vehicle	27.9	0.179
Live in Ilala	26.5	0.288
Having help with housework	82.7	0.886
Partner ANC attendance	64.8	0.045
Escorted by family member to ANC	52.8	0.049
Awareness of GDM	45.0	< 0.001
Health worker communication	27.7	< 0.001

Key: *ANC* Antenatal care, *GDM* Gestational Diabetes Mellitus

**Table 3:** Univariable analysis of risk factors for Gestational Diabetes Mellitus among women attending Antenatal care at Muhimbili National Hospital and Aga Khan Hospital Dar es Salaam in 2018

Risk factor	Prevalence (%)	*p*-value
Grand multiparity	6.9	0.709
History of GDM	1.1	0.094
History of PIH	9.5	0.022
Hypertension detected in current pregnancy	2.5	0.707
Pre-diabetic state	2.0	0.325
Glycosuria	3.6	0.002
BMI > 25	88.5	0.619
History of big baby	12.6	0.023
LGA in current pregnancy	4.5	0.903
Excessive weight gain	65.9	0.281
Family history of DM	29.6	0.671
Family history of HT	43.6	0.123
History of multiple pregnancy	3.7	0.479
History of hypertension	4.5	0.662
History of pregnancy loss	27.9	0.587

Key: *PIH* Pregnancy Induced Hypertension, *GDM* Gestational Diabetes Mellitus, *ANC* Antenatal care, *HT* Hypertension, *LGA* Large for Gestational Age, *BMI* Body Mass Index

**Table 4:** Multivariate analysis of factors associated with screening for Gestational Diabetes Mellitus among ANC attendees at Muhimbili National Hospital and Aga Khan Hospital Dar es Salaam in 2018

Characteristic	Screened for GDM		Odds ratio (95% CI)	*p*-value
	Yes = 107 (N/%)	No = 251 (N/%)		
**Glycosuria**				
Yes	9 (8.4%)	4 (1.6%)	0.96 (0.880–1.053)	0.401
No	97 (90.7%)	247 (98.4%)		
**History of delivering big baby**				
Yes	20 (18.7%)	25 (10%)	2.15 (1.127–4.101)	0.020
No	87 (81.3%)	226 (90%)		
**History of PIH**				
Yes	16 (15%)	18 (7.2%)	2.34 (1.131–4.827)	0.021
No	91 (85%)	233 (92.8%)		
**Awareness of GDM**				
Yes	67 (62.6%)	94 (37.5%)	2.79 (1.749–4.459)	< 0.001
No	40 (37.4%)	157 (62.5%)		
**Partner attendance of ANC**				
Yes	77 (72%)	155 (61.8%)	0.96 (0.902–1.038)	0.360
No	29 (27.1%)	96 (38.2%)		
**Family member attendance of ANC**				
Yes	65 (60.7%)	124 (49.4%)	1.57 (0.993–2.504)	0.053
No	42 (39.3%)	127 (50.6%)		
**Health worker communication about GDM tests**				
Yes	91 (85%)	8 (3.2%)	1.03 (0.996–1.068)	0.087
No	15 (14%)	219 (87.3%)		

Key: *PIH* Pregnancy Induced Hypertension, *GDM* Gestational Diabetes Mellitus, *ANC* Antenatal care

## Discussion

In this study the magnitude of screening for GDM was only 30%. This value is somewhat similar to a Cameroon study which found the screening level at 22% [11]. Both of these values are lower than the WHO target for a screening strategy which is 70% of the target population [16]. The study was done in specialist clinics in tertiary facilities, with adequate facilities for screening. In this study specialist clinics were considered because obstetricians were expected to be able to address the complexity and interpretation of different guidelines and tests for GDM better than other cadres. The low screening rate could not be attributed to poor resources or expertise. Another study in the region showed that health workers omit certain practices stipulated in ANC guidelines. Inadequate screening was attributed to limited resources and expertise in the rural settings in that study [7]**.**

The tests used for screening for GDM in order of frequency were RBS, FBS, HbA1C and OGTT. The trend reflects the ease of performing a particular test. Compared to the OGTT the other tests are easy to use, less labour intensive and more accessible [17]. Random blood sugar and HbA1C also do not require fasting [18]. Performing these tests is therefore convenient to both the health worker and the pregnant woman [18]. Whereas the OGTT has better diagnostic accuracy than the other tests, it is more expensive and laborious to perform [18]. This possibly is the reason why it is performed less frequently (3.4%) than other tests. If we were to use the generally accepted OGTT as the screening test for GDM, the magnitude of screening of 3.4% is far below the expected standard of care. In Uganda the uptake of the OGTT was shown to be 75.4% [5]. It would therefore plausible for health workers to offer the OGTT more in this setting.

The prevalence of undiagnosed GDM was 28%. Prevalence of GDM in a previous Tanzanian study also done in an urban setting was 8.4% [19]. Just like in our study the 75 g OGTT was used for diagnosis in that study. Our findings may be suggestive of an increasing prevalence of GDM in Tanzanian urban areas. The prevalence in our study is somewhat similar to 31.9% in Uganda [5], 31% in Cameroon [11] and 25.8% in south Africa [20]. These findings show a higher burden than the global prevalence of 16% [21]. Uptake of the OGTT was 27% which was similar to the 22% of the Cameroon study. However the uptake varies from 75.4% found in a Ugandan study [5] and 55.4% in a South African study [20]. These higher uptake rates could be because in these studies, the primary objective was to determine the prevalence of GDM using the OGTT. In our study we offered the OGTT as part of a secondary objective.

Factors found to be statistically significantly associated with screening were awareness about GDM, history of pregnancy induced hypertension (PIH) and history of delivering a macrosomic baby. Awareness of GDM in our study was 45%, which is similar to 40.3% in a multiethnic Australian study [22] but varies from 17.5% in an Indian study [23]. Similar to the findings in our study, another study in Tamil, India showed that knowledge of GDM promotes GDM screening [12]. People are more inclined to accept or do tests if they are aware about the condition and its complications. Health literacy enables patients to access and effectively use health information [24], while low health literacy is associated with poor health outcomes [25]. PIH is a mild form of pre-eclampsia and eclampsia which is the leading direct cause of maternal death in Tanzania [26]. This could explain why clinicians are more vigilant with these women. Delivering a macrosomic baby is the commonest complication of GDM in some settings. For example in a Ugandan study, macrosomia was the only obstetric complication associated with hyperglycemia in pregnancy [5]. This also explains the possible increased vigilance with these women.

## Conclusions

In conclusion, in this study we found a low magnitude of screening for GDM (30%). This is inadequate compared to the recommended WHO target of 70%. A significant proportion (28%) of women with GDM had not been diagnosed with the condition; which could account for significant adverse pregnancy outcomes.

A majority of participants (over 75%) had attended ANC 4 or more times by the time we evaluated them for screening. Those many contacts with a health worker are a missed opportunity to screen for GDM and to provide quality, comprehensive ANC services. A sizeable proportion of women were not aware of GDM. This calls for wider sensitization on GDM. Universal rather than selective screening for GDM should be adopted in this setting given the high prevalence of risk factors for GDM. A less cumbersome gold standard test for GDM diagnosis and screening needs to be developed to facilitate ease of screening. A study of attitudes and practices of ANC providers would dissect further why adequate screening for GDM is not conducted. Studies should be done to determine factors associated with poor uptake of the OGTT in this setting. Follow up studies should be done on interventions to increase screening for GDM.

### Generalizability

This study was hospital based and urban based. Therefore the study findings may not be generalised to all settings.

### Limitations

In our study, patients were consecutively sampled. This method of sampling could have introduced some level of bias in our findings. However, we have no reasons to believe that manner of selecting our sample would be associated with the dependent variable of being screened or not. The principal investigator and the research assistants were not part of the clinical teams offering ANC services.

Observational studies, like ours are prone to social desirability phenomenon while getting responses from study participants. However, the impact of this phenomenon on our study was reduced for most of the time by corroborating the participants’ responses with data that was written on the ANC. Our study did not explore the specific laboratory methods of performing the tests used in GDM screening. However, this could not have affected the study results.

Our study did not explore effects of health worker workload, knowledge and cost of test on the magnitude of screening for GDM. However, cost for the tests was either covered by insurance, heavily discounted or offered free of charge by the health services. All ANC was offered by obstetricians who are assumed to have adequate knowledge and similar level of training. We have no reason to believe that the study results could be different if these factors were explored.

## Supplementary information

**Additional file 1.** Questionnaire.

**Additional file 2.** Data extraction sheet.

## Data Availability

The datasets analysed during the study are available from the corresponding author on reasonable request.

## Abbreviations

ANC: Antenatal care
AKU: Aga Khan University
BMI: Body Mass Index
CI: Confidence Interval
FBS: Fasting Blood Sugar
GDM: Gestational Diabetes Mellitus
HbA1C: Glycated Haemoglobin
OGTT: Oral Glucose Tolerance Test
OR: Odds Ratio
PIH: Pregnancy Induced Hypertension
RBS: Random Blood Sugar
WHO: World Health Organization
