# Supplementary material for: Magnitude of screening for gestational diabetes mellitus in an urban setting in Tanzania; a cross-sectional analytic study
Source: BMC Pregnancy Childbirth. 2020 Jul 23;20:418. doi: 10.1186/s12884-020-03115-3 (PMC7379358; doi:10.1186/s12884-020-03115-3)
Supplement: Supplementary file 1 — Additional file 1. Questionnaire. [file 12884_2020_3115_MOESM1_ESM.docx]

**Additional file 1: Questionnaire (In English)**

**HOSPITAL FILE NUMBER:…….……... QUESTIONNAIRE NUMBER:………….**

**TELEPHONE NUMBER:…………………..**

**PART I: QUESTIONS TO THE PARTICIPANTS**

**FILL IN OR TICK AS APPROPRIATE** √

**SECTION I: PERSONAL INFORMATION**

1. **Age:** ………
2. **Marital status**: Cohabiting □ Married□ Separated □ Divorced □ Single□ Other □
3. **Race:** Black African□ Indian□ Arab□ Caucasian □ Chinese□ Mixed race□ Others□
4. **Occupation**: Paid employment □ Peasant □ Business woman □ Others □ Unemployed□
5. **Education status:** None □ Primary □ Secondary □ Tertiary education □ Post graduate □
6. **Do you have medical insurance?** Yes □ No□
7. **Who decided when you should start ANC?**

Yourself □ Partner□ Parent/s □ Inlaws □ Others □

1. **Residence in DSM(District):** Kinondoni□ Ilala□ Ubungo□ Temeke□ Kigamboni□ Other □ Outside Dar□
2. **Mode of transport?** Own car □ Family car □ Private hire car□ Public transport□
3. **How long does it take you to travel from your home to hospital?**

< 30 minutes □ <1 hour □ > 1 hour □ > 2 hours □ > 3hours □

1. **Do you have a family member to accompany you to hospital?**

Yes□ No□

1. **Has your husband accompanied you for ANC during this pregnancy?**

Yes□ No□

1. **Do you have somebody to help you do house chores?** Yes□ No□

**BODY MEASUREMENTS**

1. **Weight in Kilograms**: ……….
2. **Height in metres**: ………..
3. **Blood pressure:** ……………….

**SECTION II: HISTORY OF PREGNANCY**

1. **LNMP**: ………….
2. **How many times have you been pregnant?** .................
3. **History of glucose in urine in current pregnancy?** Yes□ No□ I don’t know □
4. **Previous diagnosis of pre-diabetes, impaired glucose intolerance or impaired fasting glycaemia?** Yes□ No□
5. **History of multiple pregnancies in previous pregnancies**

Yes□ No□

1. **How many times have you attended ANC in this pregnancy before today?** …………
2. **Were you checked for Diabetes Mellitus in this pregnancy with a blood test?**

Yes□ No□ I don’t know □

1. **If yes; At how many weeks of gestation was the test done?** ………….
2. **Were you told your results if you were tested for GDM?** Yes□ No□ **If Yes Results………..**

1. **Number of pregnancies lost**: ……………………
2. **Weeks of gestation (or months) at which you lost the pregnancy** ………
3. **History of pregnancy induced hypertension**? Yes□ No□
4. **History of baby’s weight ≥ 4kg in previous pregnancy** Yes□ No□
5. **History of gestational diabetes mellitus in previous pregnancies?**

Yes□ No□ I don’t know □

**SECTION III: ULTRASOUND SCAN INFORMATION**

1. **Ultra sound scan report present?**

Yes□ No□

1. **Weight on ultrasound ≥ 4kg?**

Yes□ No□

1. **Conclusion of fetus described as large for gestation in current pregnancy?**

Yes □ No□

**SECTION IV: PAST MEDICAL HISTORY**

1. **Have you heard about gestational diabetes before?** Yes□ No□
2. **History of hypertension before current pregnancy?** Yes□ No□
3. **Before you got pregnant do you think you had gained ≥ 5 kg since you were 18 years old?** Yes□ No□ I don’t know□

**SECTION V: PARTICIPANT’S FAMILY MEDICAL HISTORY (1^st^ degree relatives i.e brother, sister,mother,father, maternal or paternal aunt, uncle, grandparents)**

1. **History of hypertension/high blood pressure?** Yes□ No□
2. **History of diabetes mellitus?** Yes□ No□

**Questionnaire (In Swahili)**

**KICHWA CHA HABARI: MAGNITUDE OF SCREENING FOR GESTATIONAL DIABETES MELLITUS IN AN URBAN POPULATION IN TANZANIA.**

**NAMBA YA HOSPITALI:…….……... NAMBA YA UTAFITI:………….**

**NAMBA YA SIMU:…………………..**

**PART I: MASWALI KWA WASHIRIKI**

**JAZA AU WEKA ALAMA YA** √ **PANAPOHUSIKA**

**SECTION I: HABARI YA BINAFSI**

1. **Umri:** ………
2. **Hali ya Ndoa**: Mnaishi pamoja □ Umeolewa□ Kutengana□ Talaka□ Mjane□ Nyingine □
3. **Asili ya mtu:** Mwaafrika□ Mhindi□ Mwarabu□ Mzungu□ Mchina□ Mchangayiko□ Nyingine□
4. **Ajira**: Umeajiriwa□ Mkulima□ Biashara □ Nyingine□ Sina ajira□
5. **Elimu**: Hakuna: □ Shule ya msingi□ Shule ya Sekondari□ Chuo□ Uzamili□
6. **Una Bima ya Afya?**

Ndio□ Hapana□

1. **Nani aliamua uanze kiliniki ya wamama waujauzito?** Wewe mwenyewe□ Mwenza wako□ Mzazi/Wazazi □ Mkwe/ Wakwe zako/ □ Wengine□
2. **Unapoishi (DSM):** Kinondoni□ Ilala□ Ubungo□ Temeke□ Kigamboni□ Pengine □ Nje wa Dar□
3. **Umetumia usafiri gani?** Gari yako□ Gari ya familia□ Gari binafsi□ Gari za umma □
4. **Unatumia muda gani kutoka nyumbani mpaka hospitalini?**

< Nusu saa □ <saa 1 □ >lisali 1 □ > masaa 2 □ >masaa 3 □

1. **Je una mtu katika familia yako anayekusindikiza hospitalini?** Ndio□ Hapana
2. **Je mume wako amewahi kukusindikiza kliniki ya mama wajawazito?**

Ndio□ Hapana□

1. **Je una mtu wa kukusaidia kazi za nyumbani?** Ndio□ Hapana□

**VIPIMO VUA UKUAJI**

1. **Uzito kwa kilogramu**: ……….
2. **Urefu kwa mita**: ………..
3. **Blood pressure:** ……………….

**SECTION II: TAARIFA KUHUSU UJAUZITO HUU**

1. **Siku ya kuanza(tarehe) ya hedhi yako ya mwhisho**: ………….
2. **Hii mimba ya ngapi**? .................
3. **Sukari kwenye mkojo wakati wa mimba hii?** Ndio□ Hapana□ Sikumbuki/sifahamu □
4. **Umewahi kuambiwa kuwa unakaribia kupatwa na kisukari?**

Ndio□ Hapana□

1. **Umewahi kupata watoto mapacha?**

Ndio□ Hapana□

1. **Umehudhuria klniki ya wajawazito mara ngapi kabla ya leo?** …………
2. **Umewahi kufanyiwa kipimo cha kisukari kwa kupimwa damu?**

Ndio□ Hapana□ Sijui□

1. **Kama Ndio; Ulipimwa ukiwa na umri gani wa mimba?** …………….
2. **Uliambiwa matokeo ya kipimo?** Ndio□ Hapana□ **Kama Ndio Maajibu………..**

1. **Mimba zilizoharibika**: ……………………
2. **Umri wa mimba zilizoharibika**: ………………………………………………
3. **Historia ya pressure wakati wa ujauzito**? Ndio□ Hapana□
4. **Kuzaa mtoto mwenye kilo 4 na zaidi:** Ndio□ Hapana□
5. **Historia ya kisukari cha mimba katika mimba zilizopita?** Ndio□ Hapana□ Sijui□

**SECTION III:TAARIFA KUHUSU KIPIMO CHA ULTRASOUND**

1. **Ripoti ya Ultrasound unayo?**

Ndio□ Hapana□

1. **Uzito wa mtoto kwenye Ultrasound wa kilo 4 na zaidi:**

Ndio□ Hapana□

1. **Ripoti ilionyesha uzito wa mtoto kuwa mkubwa kuliko kawaida?**

Ndio□ Hapana□

**SECTION IV: TAARIFA KUHUSU AFYA KABLA YA MIMBA HII**

1. **Umeshawahi kusikia kuhusu ugonjwa wa kisukari mimba?**

Ndio□ Hapana□

1. **Ulingundulika na shinikizo la damu kabla ya mimba hii?:** Ndio□ Hapana□
2. **Tangu uwe na miaka 18 unajihisi umeongezeka kilo 5 na zaidi?**

Ndio□ Hapana□ Sijui□

**SECTION V: TAARIFA KUHUSU AFYA YA NDUGU ZAKO WA KARIBU UPANDE WA FAMILIA YAKO (i.e baba, mama, dada, kaka, bibi, babu, mjomba, shangazi)**

1. **Historia ya shinikizo la damu?** Ndio□ Hapana□
2. **Historia ya kisukari?** Ndio□ Hapana□
