# Supplementary material for: Magnitude of screening for gestational diabetes mellitus in an urban setting in Tanzania; a cross-sectional analytic study
Source: BMC Pregnancy Childbirth. 2020 Jul 23;20:418. doi: 10.1186/s12884-020-03115-3 (PMC7379358; doi:10.1186/s12884-020-03115-3)
Supplement: Supplementary file 2 — Additional file 2. Data extraction sheet. [file 12884_2020_3115_MOESM2_ESM.docx]

**Additional file 2: DATA EXRACTION SHEET**

*(Information to be extracted from ANC cards and laboratory test reports by researcher/research assistants)*

GRAVIDITY:……………. PARITY:…………

GDM SCREENING DONE: Yes□ No□

TEST USED TO SCREEN FOR GDM: *(tick test used for screening indicated on ANC card)*

1. FBS□
2. RBS□
3. HBA1C□
4. URINE DIPSTICK□
5. OGTT□
6. 2HR POST MEAL□
7. N/A□

TEST RESULT: ……………

WEEKS OF AMENORRHOEA AT WHICH TEST WAS DONE:………………

GLYCOSURIA: YES□ NO□

RISK FACTORS: *(tick risk factors recorded on ANC card)*

- BMI > 25 kg/M2 □
- Previous history of GDM □
- Glycosuria □
- Previous big baby □
- Poor obstetric history □
- Family history of DM □
- Known impaired glucose tolerance/impaired fasting glucose or grand multipara □

ANC done by: Nursing officer□ Resident Medical officer□ Resident doctor□ Specialist doctor □

Results of Study OGTT: Fasting ………… (mmol/l) 2 hour …………… (mmol/l)
